# Assessing Yellow Fever Risk in the Ecuadorian Amazon

**DOI:** 10.4103/0974-777X.49188

**Published:** 2009

**Authors:** Ricardo O Izurieta, Maurizio Macaluso, Douglas M Watts, Robert B Tesh, Bolivar Guerra, Ligia M Cruz, Sagar Galwankar, Sten H Vermund

**Affiliations:** *Department of Global Health, College of Public Health, University of South Florida, Tampa, Florida, USA*; 1 *Department of Epidemiology and International Health, School of Public Health, University of Alabama at Birmingham, Birmingham, Alabama, USA*; 2 *Department of Pathology, Center for Tropical Diseases, University of Texas Medical Branch, Galveston, Texas, USA*; 3 *Ecuadorian Armed Forces Hospital HG-1, Quito, Ecuador, USA*; 4 *Department of Global Health, College of Public Health, University of South Florida, Tampa, Florida, USA*; 5 *Institute for Global Health, School of Medicine, Vanderbilt University, USA*

**Keywords:** Disease outbreak, Ecuador, Military personnel, Risk factors, Yellow fever

## Abstract

This study reports results of a cross-sectional study based on interviews and seroepidemiological methods to identify risk factors for yellow fever infection among personnel of a military garrison in the Amazonian rainforest. Clinical symptoms and signs observed among yellow fever cases are also described. Humoral immune response to yellow fever, Mayaro, Venezuelan equine encephalitis, Oropouche, and dengue 2 infection was assessed by evaluating IgM and IgG specific antibodies. A yellow fever attack rate of 13% (44/341, with 3 fatal cases) was observed among military personnel. Signs of digestive track bleeding (14.6%) and hematuria (4.9%) were observed among the yellow fever cases. In 32.2% of the cases, we measured high levels of serum glutamic oxaloacetic transaminase and serum glutamic pyruvic transaminase with maximum levels of 6,830 and 3,500, respectively. Signs of bleeding or jaundice were observed in some cases, and high levels of transaminases were seen. The epidemiological and laboratory investigations demonstrated that the military personnel were affected by a yellow fever outbreak. The association between clearing the rainforest and also being at the detachments with yellow fever infection confirms that clearing is the main factor in the jungle model of transmission, which takes place deep in the Amazonian rainforest.

## INTRODUCTION

Yellow fever continues to be a public health problem in the Americas. In 1942, Brazil reported the last outbreak of this disease in an urban population;[1] nevertheless, the presence of six confirmed urban cases in Santa Cruz, Bolivia, between 1997 and 1998 evidences the re-emergence of yellow fever in urban settings of South America.[2,3] The jungle transmission of this flavivirus has been regularly reported in the Amazonian Basin during the last 30 years.[4] In the South American rainforest, yellow fever is maintained by epizootics with periodic outbreaks in human populations. Although an effective vaccine has been available for some time, countries like Brazil, Colombia, Ecuador, Peru and Bolivia maintain a constant report of cases from the Amazon Basin because of economic factors, the absence of official immunization programs against yellow fever, or both.[5–7] The Weekly Epidemiological Record published by the Pan American Health Organization reported 111 jungle yellow fever cases in 2004 and 117 cases in 2005 in the South American region, with a CFR of 45% and 44.4% respectively.[8,9]

In Ecuador, urban yellow fever was eradicated from cities in 1919. In June of 1949, jungle transmission of yellow fever was first reported among oil exploitation workers in Pastaza province[10] and periodic outbreaks have continued until the present. The last jungle outbreak was reported in 1992 in new farming settlements in the Napo and Pastaza provinces, describing 19 YF cases confirmed by serology and/or biopsy and 31 suspected cases based on their clinical signs and symptoms.[11]

The jungle pattern of YF transmission involves primarily wild vertebrates and vectors, with man becoming an accidental host. The jungle transmission cycle in the Amazon Basin is mainly maintained by *Haemagogus* and *Sabethes spp.* mosquitoes that live primarily in the crowns of trees.[7,12–14] A study of jungle yellow fever vectors carried out in the State of Amazonas, Brazil, identified *Aedes fulvus* as a selvatic vector possibly involved in the jungle transmission of yellow fever. [15] Recurrent epizootics occur in *Alouatta* (howler) monkeys, causing a high mortality rate among these primates. These phenomena confirm the observation made by Balfour in 1914, describing the “silent forest”, where all howler monkeys had died, denoting the presence of yellow fever.[16]

Factors that can determine the resurgence of human yellow fever in the rainforest include the presence of the virus; an increase in the vector population; duration of the rainy season; humidity and temperature; and colonization with an absence of preventive strategies.[17] In the Amazonian Basin, the process of colonization is related to human migration where immunologically virgin populations, like colonizers, have been the main group affected. Based on these observations, it is suggested that yellow fever is acquired by engaging in woodcutting activities.[16] Concomitantly, autochthonous populations may maintain endemic levels of the disease.

In July and August of 1997, an outbreak of hemorrhagic fever was detected among military personnel detached to the Amazonian rainforest at the Peruvian border during the armed conflict between Ecuador and Peru. A multidisciplinary team was mobilized by the Ecuadorian Armed Forces to investigate and control the outbreak.

The few studies carried out to identify risk factors for yellow fever in South America have been based on case series reports and entomological studies. This study constitutes the first systematic evaluation of risk factors during an outbreak in South America and confirms the suggested mechanisms of transmission in the Amazonian rainforest. The objectives of this report were to identify the risk factors for yellow fever infection among the military personnel affected by the outbreak, and to describe clinical symptoms and signs among yellow fever cases.

## MATERIALS AND METHODS

### Study population

The jungle garrison under study consisted of 348 subjects detached in one main post, 3 detachments, and 5 outposts, all located in the Amazonian rainforest near the Peruvian border. From the total population, seven subjects were not included because of previous vaccination against yellow fever. The geographic area was humid tropical forest, located at 100 meters above the sea level. The province population was estimated to be 57,000 inhabitants and the average population density estimated as 1.92 inhabitants per square kilometer.

### Study procedures

A cross-sectional seroepidemiological survey was conducted among the study population. After providing written informed consent, study subjects participated in a questionnaire interview concerning demographic variables, medical history and potential risk factors. In a few cases, because of the severity of the patients’ conditions, recent medical history data were obtained at the Military Hospital No.1 of Quito (HG-1) rather than from direct patient interview.

Blood samples were processed immediately after collection and sera were stored frozen at −20°C until transported on dry ice to the U.S. Naval Medical Research Institute Detachment (NAMRID) in Lima, Peru for viral isolation and serologic testing. The Ecuadorian Armed Forces and the U.S. Navy Guidelines for the use of human subjects were followed. All procedures followed international guidelines for research on human subjects and were supervised by the Ecuadorian National Council against Hemorrhagic Fevers, complemented by health officers representing the Ecuadorian Armed Forces and the Ministry of Public Health.

### Serology

Confluent monolayers of LLCMK_2_ or Vero cells were infected with prototype dengue type 1 (DEN-1), Oropuche (ORO) Peru 1992,[18] yellow fever 17D, or Venezuelan equine encephalitis (VEE) subtype I-AB virus, VR-69 (American Type Culture Collection ATCC) and Mayaro (MAY, TR467) strains. The resulting supernatant viral antigens were used to test sera for IgM antibody by a capture enzyme linked immunoadsorbent essay (ELISA),[19] and for preparing lysate viral antigens for performing IgG antibody ELISA.[20,21] A capture ELISA using goat anti-human IgM (Tago, Camarillo, CA, U.S.A.) bound to 96 well Limbro microtiter plates was used to test for IgM antibodies.[19] An indirect ELISA employing viral infected or uninfected cell lysates bound to 96 well microtiter plates was used to test serum for IgG antibody.[22]

Sera were tested initially at 1:100 dilution, and reactive samples were further tested at a 1:200 through 1:12,800 dilutions to determine the antibody endpoint titer. Virus-specific IgM and IgG antibody-positive and negative controls (run at a 1:100 dilution) were included in each test to validate the results. A horseradish peroxidase (HRP, Kirkegard and Perry, Gaitherburg, MD, U.S.A.) conjugated anti-mouse IgG and an enzyme substrate 2,2-azino di (3-ethyl-benzthiazoline) sulfonate (ABTS) were used to detect IgM antibody. HRP-conjugated goat anti-human IgG and ABTS were used to detect IgG antibody. The absorbance was read with a spectrophotometer at 414- nm wavelength. The absorbance values for the mock antigens were subtracted from those of the viral antigen to yield corrected absorbance values. Serum dilutions with corrected absorbance values greater than the reference cut-off value, estimated as the mean absorbance of 10 antibody negative serum samples, plus 3 standard deviations, were considered to be antibody positive. Samples with antibody titers of 1:200 or greater were classified as positive. All antibody titers were expressed as the reciprocal of the highest dilution yielding a positive result.

Laboratory procedures were conducted according to the principles set forth by U.S. federal guidelines in the “Guide for the Care Use of Laboratory Animals” (Institute of Laboratory Animal Resources, National Research Council, DHHS, Publication No. NHI 86-23, 1985).

The specificity of antibody reactivity was determined by a standard plaque reduction neutralization test (PRNT) using Vero cell cultures. A sample of yellow fever ELISA IgG antibody positive sera were diluted 1:10 in Earle's minimum essential medium (EMEM) supplemented with 2% fetal calf serum (FCS) heat treated at 56°C for 30 minutes. After each dilution was incubated with approximately 100 plaque-forming units (PFU) of virus, aliquots of the mixtures were tested in Vero cell cultures propagated in 25 cm^2^ flasks. Sera that reduced the viral PFU dose by 50% or greater were considered positive for yellow fever antibody.

### Virus isolation and identification

Serum samples obtained from the participants were diluted 1:5 in EMEM supplemented with 5% FCS, 200 ug/ml of streptomycin and 200 U/ml of penicillin. Aliquots of each diluted serum sample were assayed for virus by the newborn mouse and Vero cell culture assays.[23]

Aliquots of the original serum that yielded suspected isolates were inoculated intracerebrally into 1-to-3 day-old outbred mice in attempts to re-isolate the viruses. Mice were observed daily, and all mice that showed signs of illness or that died were stored at −70°C until brain tissue was extracted for viral identification studies.

The reference viruses used for preliminary identification of viral isolates by the standard indirect immunofluorescence (IFA) technique included VEE, subtype I-AB 69Z1; MAY, TR467; ORO TR9760 and yellow fever 17D strain, (American Type Culture Collection ATCC, Rockville, MD, U.S.A.). Viral isolate antigens and reference virus stocks were prepared from infected Vero cell cultures according to standard procedures. Aliquots of 20 μl of antigen and uninfected control cells were added to microscope slides, air-dried, and fixed in cold acetone for 30 minutes. Slides were air-dried at room temperature and stored at −70°C until used for preliminary identification of viral isolates.

### Management of cases

Immediately after the first cases were reported, two laboratory teams were transported by helicopter and installed in the outbreak foci with basic equipment to perform blood protozoan identification, chemical tests in blood, including SGOT and SGPT; urinary diagnosis for detecting albumin and blood in urine. The “jungle laboratories” became the key element in the referral of severe cases to the Internal Medicine and Intensive Care Unit of the Armed Forces Hospital No.1 located in Quito at 2,800 meters above sea level, where no vectors of yellow fever exist, to avoid the possible risk of initiating an urban cycle in Puyo and/or Guayaquil lowlands cities where the closest military hospitals were located. Symptomatic cases were closely monitored in two jungle health posts. Those patients who showed an increase in SGOT or SGPT and those with albumin present in urine were evacuated by helicopter to the Armed Forces Hospital No.1 in Quito.

### Statistical analysis

Logistic regression analysis was used to evaluate risk factors for yellow fever infection. Cases were defined as symptomatic subjects if they were positive for IgM, had IgG greater than 1:200, and those in whom yellow fever virus was isolated. Odds Ratios (OR) and 95% Confidence Intervals (CI) were calculated for each risk or protective factor and adjusted for the other factors in the model. The goodness of fit of the model was assessed using Hosmer and Lemeshow's Test.[24]

## RESULTS

During the outbreak investigation, 44 yellow fever cases and 3 casualties were reported among 341 subjects who had not been immunized prior to their detachment in the Amazonian rainforest. The attack rate was estimated at 13% (44 infected of 341 persons), with a case fatality rate of 6.8% (3 of the 44 infected). This study is restricted to the 338 survivors, who comprised 97% of the active personnel of the garrison. Most of the subjects were recruits assigned to one year of jungle training. Of these, 174 were from the coastal area, 73 were from the Andean zone, and 91 were native to the Amazonian rainforest. The onset of the first case was on July 1, 1997; the onset of the latest case was on August 5, 1997. The outbreak dissemination was rapidly controlled by a 100% immunization of the study subjects after the collection of blood samples; later the military personnel detached in the Amazonian rainforest were vaccinated against yellow fever. Three acute cases of Mayaro virus infection, three acute cases of Oropuche infection, and one case of HIV infection were detected during the investigation. *Plasmodium* in blood was detected in two cases and three controls, four with *P. vivax* and one patient with both *P. vivax* and *P. falciparum*.

Clearing of the rainforest (OR = 6.8; CI = 2.4-19.3) and assignment to the deep rainforest detachments or outposts (OR = 4.8; CI = 1.9-11.9) were significantly associated with yellow fever cases. The use of a bed net (OR = 0.9; CI = 0.2-4.2) did not showed protective effect; the use of repellents (OR = 0.4; CI = 0.2-1.1) had a marginally significant twofold protective effect [Table 1].

**Table 1:** Correlates of yellow fever cases among military personnel, July-August 1997

Risk factor	Yellow fever		Crude attack	OR^a^	Cl	*P* value
					
	Pos. Neg.	Rate %			
Region of birth						
Amazonian	7	84	8	0.7	0.3-1.9	0.4788
Coastal or	34	211	14	1.0	(ref)	
Andean					
Age					
≤19 years	1	26	4	0.8	0.4-1.6	0.4488
>19 years	40	271	13	1.0	(ref)	
Clearing the rainforest					
Yes	36	181	17	6.8	2.4-19.3	0.0003
No	5	116	4	1.0	(ref)	
Localization						
Detachment or	21	49	30	4.8	1.9-11.9	0.0007
outpost						
Garrison	20	248	8	1.0	(ref)	
Use of bed net						
Yes	38	270	12	0.9	0.2-1.1	0.0633
No	3	27	12	1.0	(ref)	

^a^Odds ratio (OR) and 95% confidence interval (CI) of the OR, from a logistic regression model including terms for all variables in the table

The laboratory tests performed on serum samples showed an increase in transaminases, urea and creatinine, and prolonged prothrombine times. In 32.2% of the cases, SGOT and SGPT showed high levels, up to 6830 and 3500, respectively. In 19.3% of the patients, an increase in total bilirubin was up, to 6.36mg/dl; one case presented with a bilirubin value of 22.35 mg/dl. PT was prolonged in 32.2% of the patients. An increase in creatinine and urea was observed in 6.45% of the cases.

Most of the cases presented with mild to moderate symptoms of yellow fever infection [Table 2]. Six cases presented with blood in feces (6 out of 41; 14.6%), two cases with blood in urine (2 out of 41; 4.9%), and two with jaundice (2 out of 41; 4.9%).

**Table 2:** Frequency and percentages of clinical symptoms and signs among personnel affected by yellow fever infection (n = 41), July-August 1997

Symptom or sign	Frequency	Percentage
Fever	41	100
Headache	31	75.6
Chills	26	63.4
Vomiting	18	43.9
Nausea	15	36.6
Abdominal pain	14	34.1
Arthralgias	14	34.1
Diarrhea	14	34.1
Myalgias	14	34.1
Back pain	12	29.9
Blood feces	6	14.6
Dark urine	6	14.6
Dispnea	3	7.3
Hematuria	2	4.9
Jaundice	2	4.9
Fainting	1	2.4

## DISCUSSION

This yellow fever outbreak demonstrates the re-emergence of epidemic yellow fever after the 1992 yellow fever outbreak in the same region.[11] The periodic behavior of such epidemics has been reported to occur every 5 to 10 years, linked to a re-establishment of susceptible monkey populations.[25,26] Most likely, this outbreak constituted a spread of the yellow fever epidemic reported by the Amazonas and Loreto Peruvian Departments in 1996-1997.[27] In the Amazonian enzootic areas, yellow fever virus is believed to move in waves along the galleries of the tributaries of the Amazon River.[28,29]

A 13% attack rate, a CFR of 6.8%, and a mortality rate of 0.88% were observed during the outbreak. Worldwide, the yellow fever case fatality rate has been reported to range from 16 to 38%.[13,30–32] In Cuzco department, Peru, a CFR of 20.4% was reported in the Amazonian region during 1998.[27] There are three possible explanations for the low CFR observed in this study: (1) the activities of research team increased the sensitivity of case ascertainment, increasing the detection of mild and moderate cases and the denominator of the CFR; (2) protective factors, such as previous exposure to dengue among recruits from the coastal area, age of the subjects, or genetic background, may have decreased the severity of the disease, thereby lowering the CFR; or (3) rapid access to intensive care at the Armed Forces General Hospital No.1 by applying a referral system with the establishment of “jungle laboratories”. The authors will publish the result of this analysis in a separate report.

Clearing the rainforest (OR=6.8; CI=2.4-19.3)and assignment to the detachments and outposts (OR=4.8; CI=1.9-11.9) were significantly associated with yellow fever infection. The few studies carried out during yellow fever outbreaks in the Amazon Basin have reported men between 15 and 34 years as the population at highest risk, suggesting that the disease is mainly linked to occupational exposure and recreational activities.[3] Outdoor activities, such as forest clearing, oil exploitation, hunting, lumbering, or road construction, have been most frequently reported in the few case series studies carried out in the South American rainforest.[17,27,33] The description of the jungle yellow fever outbreak in the state of Minas Gerais, Brazil, in 2001, reported that 93.7% of the cases worked in the rural areas adjacent to forests; 6.3% resided near sylvatic areas, and 96.9% were not previously vaccinated.[34]

In the Amazon jungle cycle, yellow fever virus is transmitted by *Haemagogus* spp., especially *Hg. janthinomis*, which breed in tree holes containing rainwater and live in the crowns of the trees.[27,35] *Hg. leucocelaenus*, a tree-hole breeding species, live more at ground level than *Hg. janthinomis* and can also be a good virus vector to humans.[7] An increase in the rainfall can determine high *Haemagogus* densities, with biting rates up to 100 per man per hour around midday, when humans are active in the forest.[25] Usually yellow fever cases peak in January, February and March, which corresponds to the rainy season in South America.[14] The presence of *Hg. janthinomis* has been described in the Colombian and Peruvian Amazon, close to the Ecuadorian border.[27,36] Primates, like *Alouatta* monkeys, act as the main vertebrate “amplifier” in the zoonotic cycle, mosquitoes being the real reservoir (females stay infected all life long and can transmit YF virus to their descendants).[37] Humans become infected when they enter the habitats of *Haemagogus* and are bitten by infected mosquitoes. Under undetermined favorable conditions for virus amplification, epizootics, then epidemics of yellow fever can emerge.

Use of bed nets was not associated with protection against yellow fever, and use of repellents showed only marginal protection. This is congruent with the diurnal behavior of *Hg. janthinomis*, whose activity peaks at approximately 13:00 hours, decreases around 16:00 hours, and ceases by 18:00 hours.[38]

Symptomatic yellow fever constitutes an acute viral infection, which can cause bleeding and damage of the hepatic parenchyma. The clinical spectrum varies from a mild or moderate febrile illness to a fulminating hemorrhagic fever; almost 90% of cases are mild or asymptomatic, and 10% progress to severe cases with a CFR of 50%.[3,39] Other authors describe two phases of the disease, separated by a remission period lasting from 12 to 24 hour; 15 to 25% of cases present the severe phase of the disease.[3] Patients recover from mild to moderate yellow fever disease; these cases present a febrile illness with myalgia, intense headache, conjunctival injection, abdominal pain, and vomit. This phase of the disease lasts 3 to 5 days and may present laboratory changes such as leucopenia with neutropenia and an increase in serum transaminases levels.[3] In severe cases, the patient presents hemorrhagic fever with nausea, vomit, flushed face, jaundice, and bleeding, with signs of a hepatic and renal failure. Jaundice is evident but not as deep as in hepatitis. Serum transaminase levels reflect the severity of the disease and start increasing 48 to 72 hours before jaundice appears. There is a heavy proteinuria with a tendency towards suppression of urine production; granular casts and hemoglobin can be found in urine with azotemia.[40,41] The patient can bleed from eyes, nose, mouth, bladder, rectum and other organs because of reduced synthesis of clotting factors and consumption coagulopathy.[3,16] The progression of these cases usually leads to deadly renal and hepatic insufficiency. In the terminal stage the patient enters a hepatic coma with signs of confusion, delirium, seizures and coma.[40,41] Some studies described a 20 to 50% mortality in patients with hepatorenal disease.[3]

The clinical symptoms and signs described by the patients and physicians during the present outbreak showed a low percentage of cases reporting the presence of blood in feces and urine. As described in other studies,[16,41,42] jaundice was present in a few cases, but was not deep. The laboratory tests that became our field monitor tool for hepatic function testing was transaminases, which were early indicators of worsening of the patients.

## CONCLUSION

Yellow fever virus was the identified agent for this hemorrhagic fever outbreak, which occurred in military personnel who were not previously immunized. It was evident that clearing of the rainforest placed military personnel in close contact with YF vectors like *Haemagogus* mosquitoes. The risk for acquiring yellow fever was higher in the detachments and outposts than in the garrison; therefore, it can be deduced that yellow fever transmission mainly takes place in deep rainforest, being expanded by the river galleries of the Amazonas tributaries. The low CFR observed during this outbreak could have been determined by a higher capacity to identify mild and moderate cases, by the innovative strategy applied to refer and manage severe cases, or by an anamnestic immune response developed from previous exposure to flavivirus.

A limitation of this study is that the research was carried out among military personnel only, and we suspect that some members of the Huaorani tribe could have been affected by this outbreak. Their nomadic life style made it difficult to locate and treat them adequately.

To control future outbreaks, we recommend the following guidelines:

1. All military personnel, regardless of their passive or active duty, rank or place of detachment should be immunized against yellow fever.
2. Civilians emigrating into the rainforest, like colonizers, oil company employees, road constructers, or loggers, should be immunized against yellow fever.
3. All infants above 6 months of age and children living in the Amazonian region should be immunized against yellow fever.
4. Enforce the request of the immunization card to all civilians and military personnel going into the Amazonian rainforest.
5. Active surveillance through seroprevalence of adult civilians should be carried out to assess their immune status against yellow fever.
